# Social buffering in horses is influenced by context but not by the familiarity and habituation of a companion

**DOI:** 10.1038/s41598-021-88319-z

**Published:** 2021-04-23

**Authors:** Claire Ricci-Bonot, Teresa Romero, Christine Nicol, Daniel Mills

**Affiliations:** 1 Animal Behaviour, Cognition and Welfare Group, School of Life Sciences, University of Lincoln, Lincolnshire, LN6 7TS UK; 2 The Royal Veterinary College, Hawkshead Lane North Mymms Hatfield, Hertfordshire, AL9 7TA UK

**Keywords:** Animal behaviour, Animal physiology

## Abstract

Social buffering occurs when the presence of one animal attenuates another’s stress response during a stressful event and/or helps the subject to recover more quickly after a stressful event. Inconsistent previous results might reflect previously unrecognised contextual influences, such as the nature of the stimulus presented or social factors. We addressed these issues in a two-part study of horses paired with familiar (16 subjects) or unfamiliar (16 subjects) companions. Each subject performed 4 tests in a counterbalanced order: novel object test (static ball)—alone or with companion; and umbrella opening test—alone or with companion. Social buffering was significantly influenced by the nature of the stimulus presented, but not by companion’s habituation status or familiarity. Importantly, the stimulus used produced differential effects on behavioural and physiological measures of buffering. A companion significantly reduced behavioural response (reactivity) in the novel object test but not in the umbrella test. However, heart rate recovered more quickly for subjects with a companion in the umbrella test but not in the novel object test. We propose that circumstances which permit greater contextual processing may facilitate demonstration of behavioural effects of social buffering, whereas buffering in response to startling events may be manifest only during post-event physiological recovery.

## Introduction

The concept of social support applies to a diversity of situations whereby an individual subject benefits, physically or emotionally, from the presence or behaviour of a companion^1^. There are two predominant, but not mutually exclusive, models relating to social support, and these make slightly different predictions. The first “direct effects” model predicts that companions will provide beneficial effects to the subject, regardless of whether the subject is stressed or not^1^. For example, in wild chimpanzees, a close social companion, i.e. a companion with whom the subject exchanges high rates of affiliative and cooperative behaviours, can help to reduce the activation of a subject’s hypothalamic-pituitary-adrenocortical (HPA) axis within a stressful context (intergroup encounters) but also throughout daily life (everyday affiliation)^2^. The second “social buffering” model focuses on discrete contexts only where the subject experiences a negative or stressful life event, and the companion has the potential to attenuate the stress response in one or more ways in these circumstances. The latter is the focus of the current paper.

There are many examples of social buffering in the literature where conspecific animals have been shown to reduce the subject’s stress response during a stressful event^3–5^. For example, marmosets exposed to novelty show fewer behavioural signs of distress and agitation, and do not have significantly increased levels of urinary cortisol if accompanied by a companion^6^. The effects of social buffering can be further distinguished according to whether the companion is present during or after the stressful event experienced by the subject. In both cases, the presence of a companion seems to have a positive impact on the subject’s response. For example, the presence of a conspecific rat can reduce behavioural signs of stress when a subject rat is exposed to a conditioned stimulus^7^. In a similar way, rats housed with a littermate after experiencing social defeat show more exploratory behaviour in an elevated plus maze test than those housed alone^8^.

The qualitative properties of stressful stimuli that have been used to examine social buffering vary from novel objects or environments to the appearance of a sudden stimulus, white noise, electric shocks, social defeat and social separation and/or isolation^5^. Similarly, measures of social buffering are very diverse, and include the return of corticosteroid levels to baseline, heart rate, body temperature and *Fos* gene expression and/or behavioural measures of vocalization, escape attempts, immobility, defecation rate and motivation to seek social contact depending on the study^3,5^. This diversity in both stressful stimuli and measurement parameters has resulted in studies with very different objectives (from assessing the social effects of anxiolytic drugs^9^ to developing animal models of post-traumatic stress disorder^10^) but such diversity also *hinders* the development of a coherent understanding of important sources of variability in the social buffering process.

This is especially apparent when trying to draw conclusions about the impact of familiarity and/or companion’s arousal state on social buffering. Indeed, contradictory results about the role of companion familiarity abound, with some studies showing familiar animals are better buffers (guinea pigs:^11,12^; marmoset:^13^; rat:^14,15^; Siberian dwarf hamster:^16,17^), while others indicate that unfamiliar animals are better (^18,19^) and some report no effect (cattle:^20^; rat:^21^). Moreover, many researchers have demonstrated that the arousal status of the companion can affect its efficiency as a social buffer (cattle:^22^; chicken:^23^; rat:^24,25^). Companions that have previously experienced or that anticipate the arrival of a stressor do not appear to be good social buffers in cattle^22^ or rats^24,25^. However, in horses^26^ and mice^27^, if companion arousal is reduced by prior habituation to a novel but harmless stimulus then this can increase the companion’s effectiveness as a social buffer.

It is reasonable to expect that some general principles govern the social buffering process, but these are probably masked by great diversity (of species, stimuli and measures taken), confounding factors (e.g. lack of standard definitions and measures of subject-partner association level) as well as gaps in our knowledge. For example, individual studies of social buffering effects have all focussed on one stressor at a time, so it is not possible to known from any individual study whether the impact of the companion on the subject’s stress response is context-dependent. To our knowledge, only one study has compared the impact of different stressors^28^. In this rare example, Takeda et al.^28^ examined the responses of groups of heifers to three different tests: a novel object test, a surprise test and a conflict test. Conspecific familiarity affected the behavioural responses in the novelty test and surprise test, and the physiological responses in the surprise test and conflict test. However, in this study the heifers were never alone, but in groups of either 2 or 5, and so this study must be considered to examine group size effects rather than factors affecting the occurrence of social buffering.

Our aim is to contribute to the development of a general model of social buffering by investigating, for the first time, whether the effectiveness of factors such as companion arousal and familiarity are influenced by stimulus context. We used horses as a study species because of their complex social system^29,30^ and increasing evidence of the sophistication of their social cognition. Horses are, for example, able to exchange information through subtle communication^31,32^ and can perceive the emotional state of their conspecifics^33^. We also used both behavioural and physiological measures to assess the extent of social buffering.

In two studies involving two different groups of subjects, we tested if a companion can attenuate the subject’s stress response. In the first study, we also tested whether the impact of companion arousal on the subject’s stress responses was context-dependent by exposing subjects to two distinct stressful events while using pairs of subjects with a high level of consistent familiarity. The hypotheses were that the companion will help to reduce the behavioural and physiological responses across different stressful contexts, and that a habituated companion will be more efficient at buffering the subject’s stress responses than a naïve one. We then evaluated in a second study whether similar effects of companion arousal and the nature of the stressful event were obtained when individuals were not familiar with their companions. The hypothesis was that a familiar companion would be more effective in reducing behavioural and physiological responses than an unfamiliar companion.

## Methods

### Ethics statement

This research was carried out in compliance with the ARRIVE guidelines. The delegated authority of the University of Lincoln Research Ethics Committee approved this research (CosREC433) and all methods were carried out in accordance with the university Research Ethics Policy and with the ethical guidelines of ISAE. Written informed consent was obtained from the owner of the horses.

### Study animals

The first study took place between May and June 2018 in the riding school of Haras de Jardy (Versailles, France). 32 horses (16 pairs of familiar horses; pairs were housed together in stables within a large building and had lived together for over 1 year) of various breeds, aged 6–24 years old (mean ± SD: 13.2 years,  ± 4.1) representing 10 geldings and 22 females which belong to a busy riding school environment. Each pair (n = 16) consisted of a focal subject and a companion and the roles were not reversed during the experiment.

The second study was undertaken at the same location as the previous experiment, from April to June 2019. 32 horses of different breeds, consisting of 16 pairs of unfamiliar horses (unfamiliar companions lived at the riding school, but were housed in a separate building from the subjects) were used, aged 4–23 years old (mean ± SD: 14.2 years,  ± 4.6) representing 19 geldings and 13 females. Among the 32 horses, 7 were used in both studies and played the role of habituated companion on both occasions. As in the first study, each pair (n = 16) consisted of a focal subject and a companion. Within a pair the roles were not reversed during the experiment.

### Experimental set-up

A rectangular test arena (6 m × 3 m) with a starting area (3m × 3m)^34^ was built with wooden poles (see Figure S1 in the electronic supplementary material). This test arena contained 2 buckets of food to avoid competition within pairs^35^, separated by 1 m (each bucket contains 600 g of pellets) with free access during the test. The distance between the buckets and the novel object or the umbrella was 1 m.

### Test stimuli

Two different types of novel stimuli were presented in a counterbalanced order to control for prior exposure effects. One was a novel unchanging stimulus (75 cm black and white striped ball, “novel object test”), the other a novel suddenly changing stimulus (opening of a blue and white 120 cm umbrella, “umbrella test”). These different stimuli were presented in front of the horses by an experimenter^36^.

### General habituation to test environment and allocation of experimental roles

Each horse was habituated individually to the test arena prior to the experiment to reduce the risk of stress due to a new environment, using the same principles of habituation described by Christensen et al.^26^. The horses were considered ready for use when they met the following criteria: the horses entered the arena voluntarily and walked directly to the bucket to eat for at least 90 s out of a total of 120 s. During this habituation process, the novel object was absent, the umbrella was closed and the person who would open the umbrella stood still behind it to habituate the horse to their presence. The umbrella and associated individual (same person for all tests) were present throughout every experiment (novel object and umbrella test).

The allocation of experimental roles (subject or companion) within a pair was done by measuring the time taken by individuals to go to the bucket and eat during the habituation period and by the number of habituations achieved per horse^26^. The horse which required fewer trials was selected as the companion, with the other horse being the subject. If the two horses required the same number of trials, then the fastest responding horse within the trials was made the companion: it was reasoned that a faster-responding horse spending less time examining its environment would be more confident and therefore potentially a better social buffer.

### Habituation of habituated companion to the different stimuli

In the first study, half of the companions (n = 8) were habituated to the test stimuli (novel object test and umbrella test), these are referred to as habituated companions. They were regarded as habituated when they met the following predefined criteria during the stimulus exposure: little (reactivity score of 1) or no behavioural reaction (reactivity score of 0) to the stimulus and no greater increase in heart rate than 20 beats/min during stimulus exposure^26^. Following Christensen et al.^26^, they underwent up to 13 trials. If they had not reached the habituation criteria after the 13th trial, they would have been dropped from the study. To ensure that they remained habituated, the procedure was repeated to the stimulus (ball or umbrella) once, less than 10 min before performing the test with the subject.

The other eight companions were not habituated to the test stimulus, they are referred to as naïve (control) companions.

In the second study, the same eight habituated companions from the first study were scheduled for use, however one horse had to be replaced because it had been retired. These companions did further habituation sessions to the test stimuli (novel object test and umbrella test), following the same procedure as before (based on Christensen et al.^26^). In addition, eight horses that did not participate previously in the study were used as naïve companions.

### Recordings

The heart rate was only recorded for the focal subjects. These were habituated to a Polar Equine H7 heart rate monitor, and heart rate data analysed with the software Kubios HRV 3.0.2. The settings of the heart rate monitor were such that the sampling occurred 1/s, with the monitor always sending the current heart rate, not the rolling average. The week before the tests, the focal subject’s heart rate was measured for two minutes in the test arena with the same conditions as in the test (i.e. when the subject was eating from the bucket) but without being exposed to any test stimuli (i.e. pre-test heart rate)^37,38^. Heart rate recovery (HRR) was measured from the time it took for the subject’s heart rate to return to within 15% of this pre-test heart rate value (bpm) after being exposed to the test stimulus^33,39^. The threshold for determining recovery of pre-test heart rate value plus 15% was chosen as it represents a substantial degree of decline and accommodates individual variability in baseline values based on the pre-test heart rate value; as a result, most horses recovered during the allocated time (5 min). If a horse escaped from the test arena in response to the presentation of the stimulus during the test, it was assigned a value of 300 s which corresponds to the maximum duration of the testing session. The heart rate data was checked for artefacts. In the analysis, all recordings with more than 5% heart beat errors were excluded (3 in the novel object test alone and 2 in the umbrella test alone for the first study)^39^.

Behaviours were recorded by two cameras during testing which had been present throughout habituation.

The behavioural responses of both subjects and companion horses to the novel object (ball) and the opening of the umbrella were evaluated by assigning a score (ranked in order of intensity: 0 = no reaction, 1 = head up, 2 = alert, 3 = away, 4 = flight and 5 = escape), called reactivity score (RS) (see Table S1 for behavioural description in electronic supplementary material)^26^. Two evaluators separately watched and rated the same videos in order to assure a reliable reporting of the behavioural data (see the electronic supplementary material S1).

### Experimental procedure

**Figure 1:**
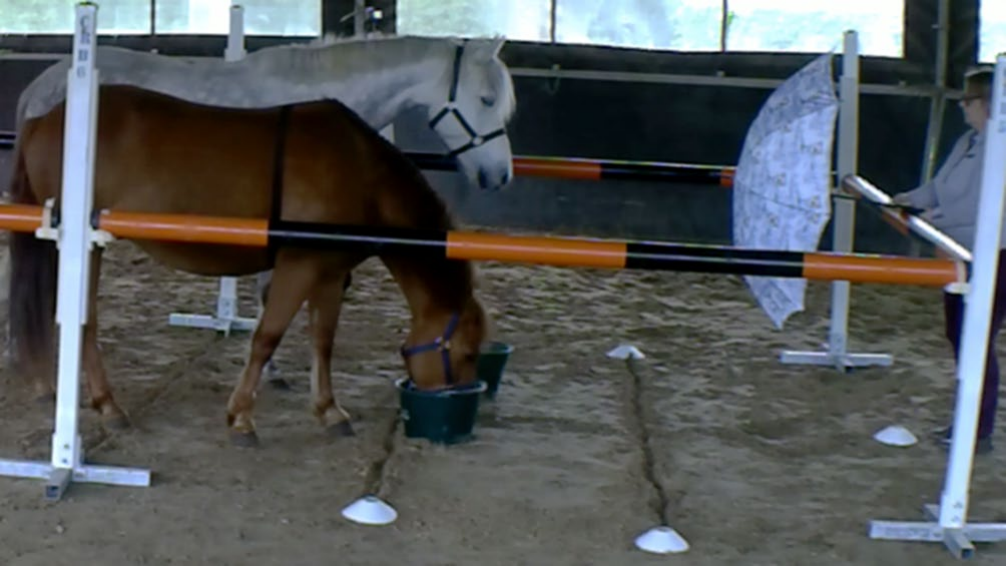
Subject (brown horse) during the umbrella test with its companion (white horse).

In order to determine if the companion could help to reduce the subject’s stress response, the subject was tested alone and with its companion for each stimulus. The tests took place between 8 am and 1 pm. Once the focal subject with or without its companion had been released into the test arena, the experimenters waited until the focal subject had eaten with its head in the bucket for 3 s before introducing the novel object or opening the umbrella in the arena^40^, the test then ran for a further 5 min. If the companion was present, it was next to the subject when the stimulus was applied. The two horses were free to interact and to eat from the buckets in the test arena (see Fig. 1). At the end of the 5 min, the horses were taken back to their housing.

Each subject performed 4 tests: novel object test (ball)—alone or with companion; and umbrella test—alone or with companion. Each horse did only one test per week. To control for order effects, the 16 pairs were randomly allocated into 4 groups; each group contained 2 pairs of subjects with habituated companions and 2 pairs of subjects with naïve companions. With each group performing the tests in a different order to control for order, the potential effect of test order was minimised, but it was still considered in the statistical model.

### Data analysis

The software R (version 3.5.2) was used for all analyses^41^.

For each study, the data from one pair was removed because the companion stayed in the starting area during the umbrella test. The companion was therefore not considered able to play the role of social buffer. For this case, only the data obtained in the novel object test alone and by pair were kept in the analyses.

For the population level data, we first we established that there was no order effect on either the behavioural or physiological responses of subjects (see the electronic supplementary material S1).

In order to verify if the habituation of the companion had an impact on their responses, the companions’ reactivity score was compared between habituated and naïve individuals across the two different situations (Mann–Whitney test). In order to compare the median values of the pre-test heart rate with the heart rate obtained when the subject was alone in the novel object and umbrella test, a Wilcoxon test was used. This determined if the horse’s heart rate was significantly higher when the stimulus was presented.

For the next stage of analysis, since our focus was on factors affecting the occurrence of social buffering, all subjects with a reactivity score of 0 when they performed the test alone were excluded from the behavioural analyses as there was no potential to show a social buffering effect on their responses. Subjects who did not show a change in heart rate (obtained 0 s recovery time) when they were alone were excluded from the physiological analyses. Exclusion for one of the analyses did not necessitate exclusion from other analyses. To examine if the companion reduced the behavioural and/or physiological responses of subjects during stressful situations, the reactivity score and the heart rate recovery were compared between when the subject was alone or with a companion for each test. Cumulative link mixed models (CLMM) using the adaptive Gauss-Hermite quadrature (AGQ) (with the R-package *ordinal*) were used for the data on reactivity score and linear mixed effect models (LMM) in the R-package *lmerTest* used for the data on heart rate recovery, with companion (absent vs. present) as a fixed factor and the identity of the subjects as a random factor.

Finally, to determine if a habituated companion had a different effect to a naïve companion, the difference in reactivity score (between when the subject was alone or with a companion) and heart rate recovery time (between when the subject was alone or with a companion) were both compared as a function of the types of companion (naïve vs. habituated) for each test. The data on reactivity score were analysed with a cumulative link model (CLM) and the data of heart rate recovery with a linear effects model (LM). Similar models were used for each dependent variable, with type of companion (habituated vs. naïve) as fixed factors. In addition, companion reactivity score was added as a fixed factor in the CLM of reactivity score.

The significance of the results was assessed at a threshold of p < 0.05.

## Results

### Study 1: impact of familiar companion

#### Influence of context

**Figure 2:**
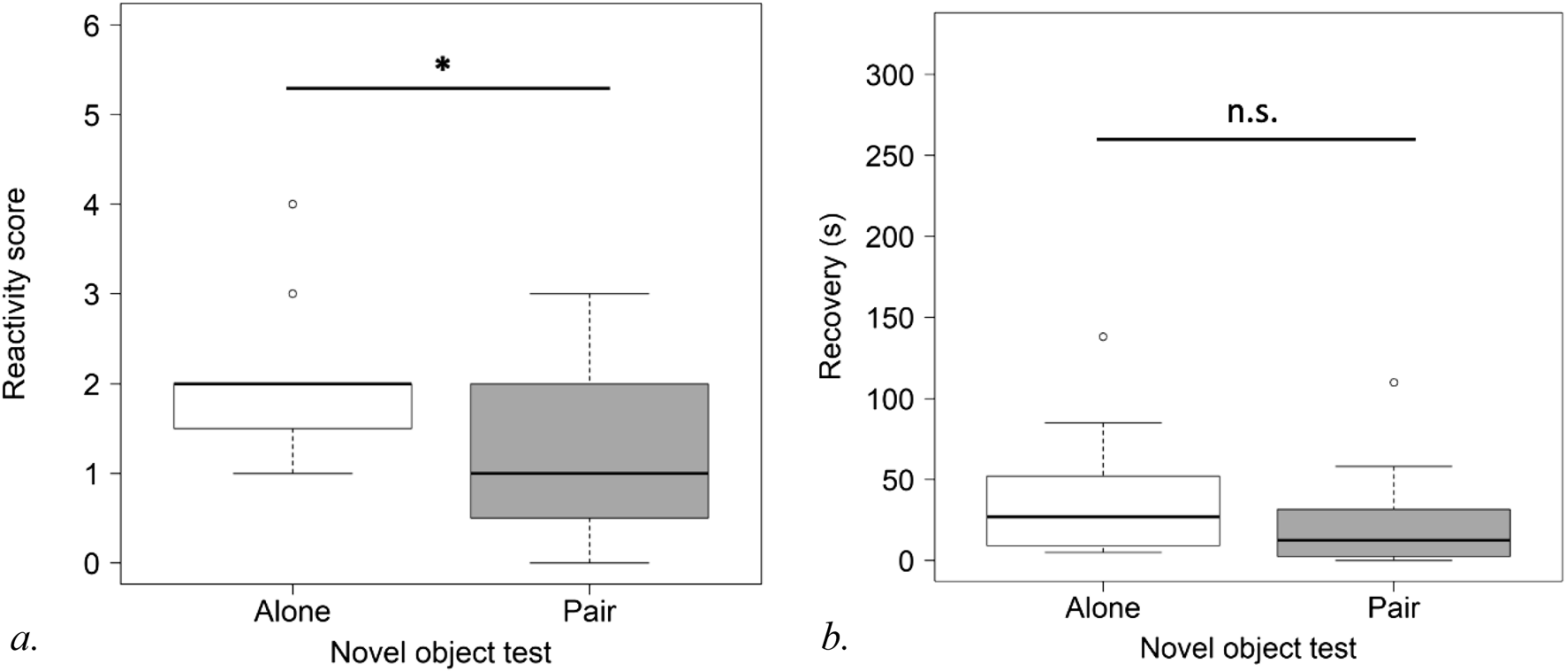
Subject’s reactivity score (**a**) and heart rate recovery (**b**) (raw values) in novel object test without and with familiar companion (*P < 0.05).

Reactivity scores were analysed for 16 pairs for the novel object test. In this test, subjects in pairs had significantly lower reactivity scores than those alone (Cumulative link mixed models (CLMM): estimate ± S.E.: − 2.12 ± 0.88, P = 0.016) (see Fig. 2a). Very few subjects investigated the ball in the novel object test (2 in the alone condition and 2 others in the condition with the companion).

The mean value of the subject’s pre-test heart rate was 42.29 (SD = 4.58) and significantly increased to 48.33 (SD = 12.54) during the novel object test (Wilcoxon tests: V = 6, n = 13, p = 0.011).

The analyses of heart rate recovery were run on 12 pairs in the novel object test. The difference in time to recover obtained from the subjects in pairs and when alone was not significantly different (Linear mixed effect models (LMM): t-value, df: − 1.336, 11, P = 0.208) (see Fig. 2b).

The companion’s reactivity score did not have an impact on the subject’s reactivity score in the novel object test (Cumulative link model (CLM): estimate ± S.E.: 0.31 ± 0.51, P = 0.535).

**Figure 3:**
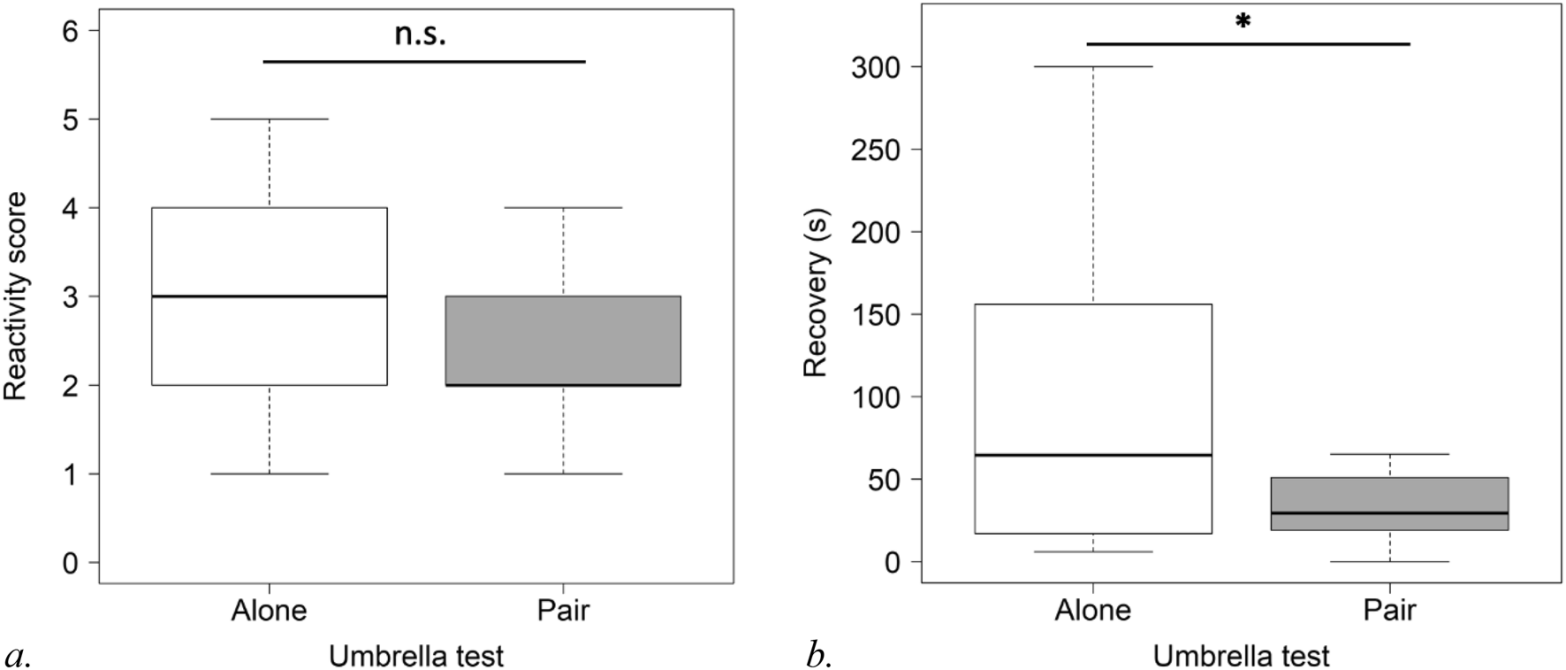
Subject’s reactivity score (**a**) and heart rate recovery (**b**) (raw values) in umbrella test without and with familiar companion (*P < 0.05).

For the umbrella test, reactivity scores were analysed for 14 pairs. Considering only subjects who showed a reaction, the reactivity score for the subjects in pairs was not significantly lower than that obtained from those alone (CLMM: estimate ± S.E.: − 1.14 ± 0.78, P = 0.145) (see Fig. 3a).

The mean value of the subject’s pre-test heart rate was 41.50 (SD = 3.66) and significantly increased to 50.68 (SD = 8.66) during the umbrella test (Wilcoxon tests: V = 7, n = 13, p = 0.004).

The data on heart rate recovery were analysed for 14 pairs for the umbrella test. The presence of a companion was associated with quicker heart rate recovery (LMM: t-value, df: − 2.319, 13, P = 0.037) (see Fig. 3b).

The reactivity score of the companion did not have an impact on the subject’s reactivity score in the umbrella test by pair (CLM: estimate ± S.E.: − 0.37 ± 0.77, P = 0.623).

#### Impact of habituation status on companion’s reactivity score

The median value of the companion’s reactivity score in the novel object test was 0.5 (Quartile 1 (Q1) = 0; Quartile 3 (Q3) = 1) for habituated companions (only one of them produced a reaction score higher than that allowed for a habituated companion) versus 1 (Q1 = 0; Q3 = 2.5) for naïve companions. These values were not significantly different (Mann–Whitney test: W = 23.5, n1 = 8, n2 = 8, P = 0.372). In the umbrella test, the median value of the companion’s reactivity score was significantly lower at 1 (Q1 = 1; Q3 = 1.5) for habituated companions compared to 2 (Q1 = 2; Q3 = 3) for naïve companions (Mann–Whitney test: W = 4, n1 = 8, n2 = 7, P = 0.004).

#### Impact of companion habituation on subject’s responses

The difference in reactivity scores and heart rate recovery were not significantly different between subjects with habituated companions and subjects with naïve companions regardless of the test (Reactivity score (RS), CLM: Novel object test (NO), estimate ± S.E.: 0.36 ± 0.95, P = 0.705; Umbrella test (UM), estimate ± S.E.: 0.20 ± 1.36, P = 0.881; Heart rate recovery (HRR), Linear effect model (LM): NO, t-value, df: − 1.027, 10, P = 0.329; UM, t-value, df: 0.216, 12, P = 0.833).

### Study 2: Impact of unfamiliar companion

#### Influence of context

**Figure 4:**
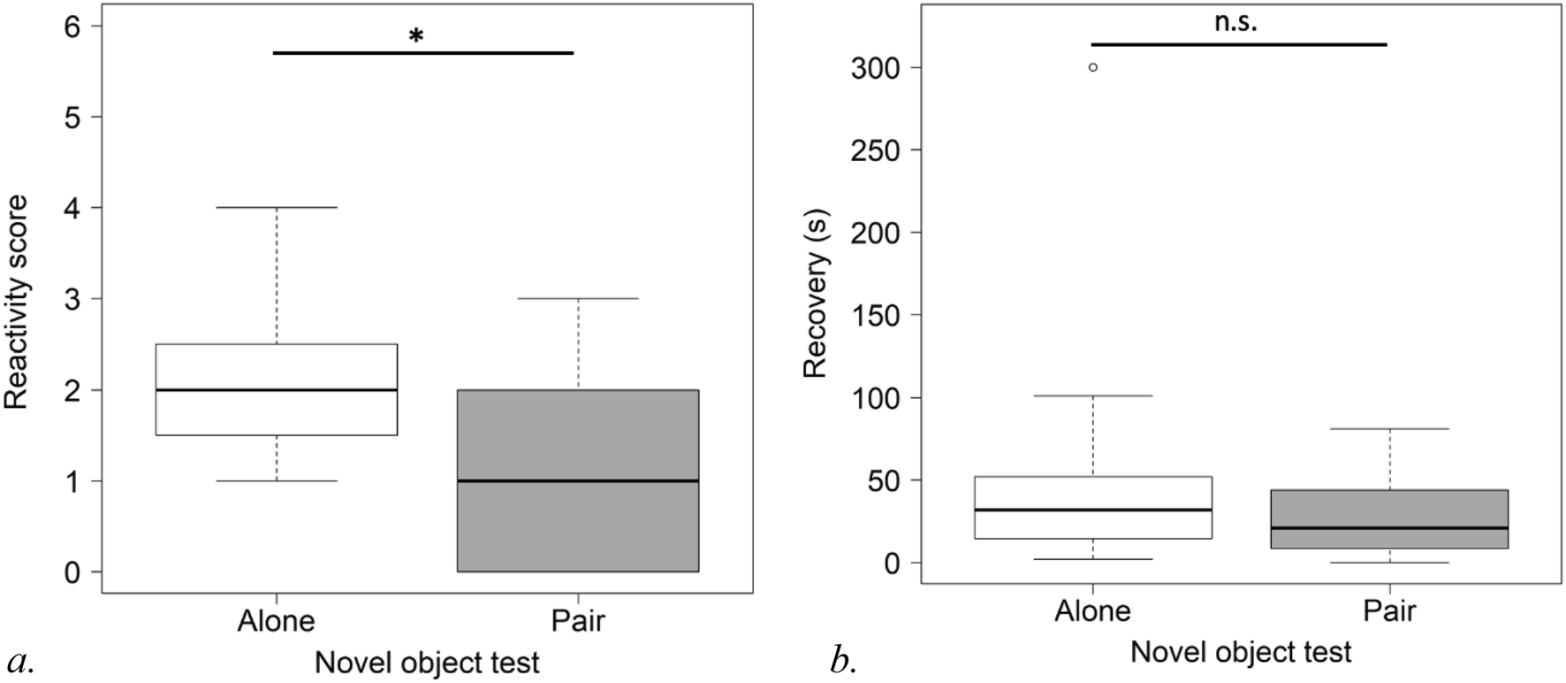
Subject’s reactivity score (**a**) and heart rate recovery (**b**) (raw values) in novel object test without and with unfamiliar companion (*P < 0.05).

For the novel object test, the analysis of reactivity scores was run on 12 pairs. The subject’s reactivity score in the novel object test for paired subjects was significantly lower than when alone (CLMM: estimate ± S.E.: − 2.87 ± 1.19, P = 0.015) (see Fig. 4a).

The mean value of the subject’s pre-test heart rate measurement was 48.34 (SD = 4.73) versus 50.53 (SD = 10.52) for the novel object test. These values are not significantly different (Wilcoxon tests: V = 56, n = 16, p = 0.552).

The data on heart rate recovery were analysed for 11 pairs in the novel object test. The time to recover for the subjects in pairs was not significantly different to those alone (LMM: t-value, df: − 1.209, 10, P = 0.254) (see Fig. 4b).

In the novel object test pairs, the companion’s reactivity score did not have an impact on the subject’s reactivity score (CLM: estimate ± S.E.: 0.07 ± 0.52, P = 0.885).

**Figure 5:**
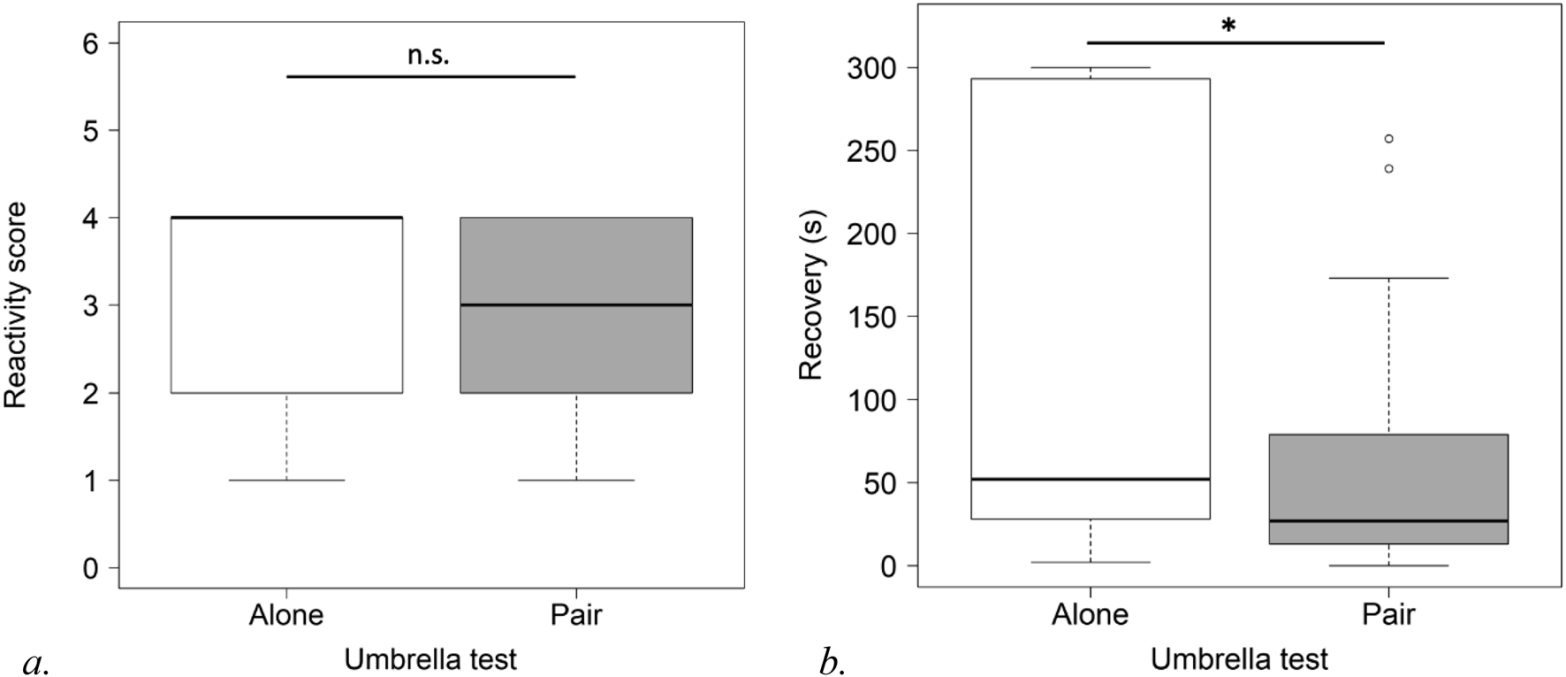
Subject’s reactivity score (**a**) and heart rate recovery (**b**) (raw values) in umbrella test without and with unfamiliar companion (*P < 0.05).

In the umbrella test, the reactivity scores were analysed for 15 pairs. The reactivity score for the subjects in pairs was not significantly lower than that obtained from those alone (CLMM: estimate ± S.E.: − 1.08 ± 0.96, P = 0.259) (see Fig. 5a).

The mean value of the subject’s heart rate pre-test measurement was significantly lower at 48.34 (SD = 4.73) versus 71.54 (SD = 33.04) during the umbrella test (Wilcoxon tests: V = 28.5, n = 16, p = 0.044).

The data on heart rate recovery were analysed for 15 pairs for the umbrella test. Subjects in pairs had significantly quicker recovery times compared to those alone (LMM: t-value, df: − 2.229, 14, P = 0.043) (see Fig. 5b).

The subject’s reactivity score was not impacted by the reactivity score of the companion in the umbrella test (CLM: estimate ± S.E.: − 0.68 ± 0.46, P = 0.137).

#### Impact of habituation status on companion’s reactivity score

The median value of the companion’s reactivity score in the novel object test was 0 for habituated (Q1 = 0; Q3 = 0; only one produced a reaction score higher than that allowed for a habituated companion) and naïve companions (Q1 = 0; Q3 = 1). These values are not significantly different (Mann–Whitney test: W = 25, n1 = 8, n2 = 8, P = 0.369).

In the umbrella test, the median value of the companion’s reactivity score was significantly lower at 1 (Q1 = 0.5; Q3 = 3) for habituated companions versus 4 (Q1 = 2.5; Q3 = 4) for naïve companions (Mann–Whitney test: W = 11, n1 = 8, n2 = 7, P = 0.047). Only one subject investigated the umbrella and this was an individual with a companion. During the tests 11 pairs had agonistic exchanges such as head threat when they ate, i.e. one of the horses turned its heard toward the other with the ears pinned back.

#### Impact of companion habituation on subject’s responses

Regardless of the test, there were no significant differences in reactivity scores and heart rate recovery, between subjects with a habituated companion and subjects with a naïve companion (RS, CLM: NO, estimate ± S.E.: 0.37 ± 1.08, P = 0.731; UM, estimate ± S.E.: 1.92 ± 1.42, P = 0.174; HRR, LM: NO, t-value, df: − 1.477, 9, P = 0.173; UM, t-value, df: 1.287, 13, P = 0.221).

## Discussion

Among the many examples of social buffering^3–5^, there is inconsistency in the conclusions drawn about general effects^42^. These relate to variability in the stimuli and measurements assessed; our work allows the development of a framework for rationalising these inconsistencies. Our results show that while the nature of the stressor produces a differential effect on behavioural and physiological measures of buffering, the characteristics of the companion such as its arousal status or familiarity, did not have a significant effect. A companion significantly reduced the subjects’ behavioural response (reactivity) in the novel object test but not in the umbrella test; the converse was found in relation to heart rate recovery time. This interaction between stimulus type and behavioural versus physiological buffering effect has not been noted before. Moreover, the consistency of the effects in the two studies show the robustness of the phenomenon.

The differential impact of the companion on the subject’s behavioural response might relate to the emotional quality and intensity of the response provoked by the two stimuli (ball or opening of the umbrella). Like others, we found a greater reaction to an umbrella than to a ball (or ball-like stimulus such as a cone e.g.^43,44^). Although theoretically, this might simply relate to the umbrella being larger, for example, Bulens et al.^44^ suggested that the large size of an opened umbrella makes it difficult to ignore compared to the cone and plastic ball used in their test. However, stimulus size alone does not seem to be a full explanation as horses have been observed to react similarly to a stationery umbrella and a stationery but larger plastic tarp (5 m × 6 m)^45^. Another possibility, evident in our study, is that stimulus movement played an important role on the subsequent response: the umbrella was a novel, suddenly changing stimulus which led to a rapid change in the environment at a given distance from the subjects, unlike the ball. The sudden change provoked a more dramatic reaction (shown by the reactivity scores in Figs. 3a and 5a) in our subjects, manifest as the natural flight responses to a direct and real threat^46^. In these potentially dangerous situations individuals would not be expected to seek further information about the situation^47^. This behavioural response is consistent with other findings that horses accept a novel unchanging stimulus more easily than a novel suddenly changing stimulus^48^. The suddenness of the change reduced the degree of control that subjects had over the umbrella stimulus. Having an effective active response in the presence of an aversive stimulus has repeatedly been shown to reduce the physiological and behavioural stress response^49^ as well as the degree of aversion experienced^50^. The difference in ability of the subject to control the level of exposure to these stimuli may influence the quality of emotional responses. The ball, is not a sudden stimulus, but might provoke a degree of uncertainty/anxiety. In this state, the animal displays increased attention and investigatory behaviours, such as smelling and licking in order to learn more about the situation with inhibition of many other behaviours^46,51^. This is an adaptive strategy as the uncertain animal potentially gains an advantage from gathering further information at little risk; it is therefore more likely to be influenced by social cues. In our research, the subjects have been tested twice with each stimulus in order to standardise the intervention (i.e. colour, shape, size, speed of appearance) and ensure that responses were directly comparable.

Heart rate recovered more quickly for subjects with a companion in the umbrella test but this was not the case in the novel object test. It is possible that in the umbrella test, the immediacy and mental focus of the startle response meant the behavioural reaction was not susceptible to social buffering; however, having moved away from the umbrella, it might be that the companion can act as a social buffer during the recovery phase. In this situation, it would be predicted that the effect of the companion would only be manifest physiologically (e.g. in heart rate recovery). In addition, it could be argued that the intensity of the emotional response (or general arousal) rather than its quality (anxiety versus fear) differentiates the two conditions. In the former case, the opening of the umbrella may have increased heart rate to a greater peak, more readily permitting a reduction by the companion, which is not the case with the novel object test. In both of our studies, the mean heart rate obtained during the 5 min of the umbrella test alone was significantly different from the pre-test heart rate measurement. The same result was found in Study 1, for the novel object test alone. By contrast, in Study 2 mean heart rate was not significantly higher than the pre-test heart rate measurement. Our result for Study 2 is consistent with that found by Takeda et al.^28^ with Japanese Black Heifers, where a weak (non-significant) effect was reported for a strange object on mean heart rate compared with a marked increase in heart rate during a surprise test (bucket dropped). However, as already noted, their study examined group size effects and not social buffering effects since there was no condition where individuals were alone. Consequently, it seems a middle stressor, such as a novel object, has a relatively small impact on a subject’s physiology which could explain the lack of physiological social buffering effect in the ball, novel object effect. The more gradual mental processing^52^ may facilitate greater social buffering of the behavioural response in these circumstances.

Thus we postulate that the ball caused a state of lower level increased arousal and possibly anxiety, while the sudden opening of the umbrella resulted in a fear state with a discernible startle response^52^. The differing social buffering effects that we noted with the two stimuli can be explained by the fact that the animal needs time to process relevant information such as a companion’s behaviours, however during sudden changes this mental process is focused on the immediate, rapid response that might be necessary to protect the individual from harm.

The arousal status of the companion (i.e. companion’s habituation or not to the stimuli) did not affect the subject’s reaction and recovery time in either of our studies. This is not surprising for the novel stimulus test, given that the behavioural response of habituated and naïve companions was similar. Indeed, only 4 horses from the first study and 2 from the second study produced a reaction score higher than that allowed for a habituated companion (and one of these horses in each study was a habituated companion). However, in the sudden stimulus test, the behavioural response was different between the two types of companion, with habituated companions showing less reaction than naïve companions. This finding is in contrast to some others who have reported that companions are more effective social buffers if their arousal is reduced by prior habituation to a novel but harmless stimulus^26,27^. Christensen et al.^27^ found that a habituated demonstrator horse reduced the fear reactions of unrelated young horses facing an opening umbrella. This difference in results between our study and that of Christensen might be explained by the age of the horses. Our horses being older were more experienced and therefore potentially more likely to have habituated to a wider range of sudden changes in the environment^53,54^. In the study of Christensen et al.^27^, the horses were just two years old, relatively inexperienced, and more likely to show flight responses. As the behavioural response is not always correlated with the physiological response^55,56^, it would be useful, in future, to record the behavioural and the physiological responses of the companion as well as the focal subjects in order to have greater insight into their arousal state^57^.

Our results also showed that in horses, familiarity between the subject and companion also had no effect on the subject’s stress responses. In the literature, the role of companion familiarity varies with factors such as the nature of the stressor and also with the species studied^5^. Some^20^ have found that after social isolation, reunion with any conspecific (familiar or unfamiliar) helped to reduce behavioural responses; while others^28^ found that a familiar companion was a more efficient buffer of the behavioural response during a novelty test and surprise test, and the physiological response during a surprise test and conflict test. The importance of familiarity between individuals seems to vary with age, potentially being less important with older subjects^20,^^58^. In adult cattle, after a separation from the group, any form of conspecific could help to reduce the subject’s behavioural responses during the reunion period^20^. However, in calves, it has been found that a familiar conspecific was more effective than an unfamiliar conspecific in reducing the behavioural response during a separation from the group^58^. The results of these studies are potentially consistent with the lack of effect of familiarity in our own study as the horses used are older (mean age 13.2 years in the first study and 14.2 in the second one). Whether or not these results apply to younger or less experienced horses needs to be investigated.

It is important to highlight that the familiarity of a subject is not the same as its relationship. Familiarity implies recognition of an individual, i.e. an individual is able to identify another whom it has previously met based on distinctive characteristics^59,60^; but does not imply any form of social bond. We suggest that future studies should investigate the potentially separate effects of social bond and familiarity on social buffering effects.

In conclusion, neither the arousal status nor the familiarity of a companion influenced the responses of adult subjects in our study. However, we found strong effects of stimulus context. We propose that when the risks of referencing another individual in a stressful situation are low, social buffering of the initial behavioural response will be evident; however when there is a high potential risk from the initial immediate response, social buffering effects will only be evident in the physiological recovery of the horse after the event.

## Supplementary Information

Supplementary Information
