# Supplementary material for: Social buffering in horses is influenced by context but not by the familiarity and habituation of a companion
Source: Sci Rep. 2021 Apr 23;11:8862. doi: 10.1038/s41598-021-88319-z (PMC8065151; doi:10.1038/s41598-021-88319-z)
Supplement: Supplementary file 1 — Supplementary Information [file 41598_2021_88319_MOESM1_ESM.docx]

# Social buffering in horses is influenced by context but not by the familiarity and habituation of a companion.

Claire Ricci-Bonot^1*^, Teresa Romero^1^, Christine Nicol^1,2^ and Daniel Mills^1^

^1^ Animal Behaviour, Cognition and Welfare Group, School of Life Sciences, University of Lincoln, Lincolnshire, LN6 7TS United Kingdom

^2^The Royal Veterinary College, Hawkshead Lane North Mymms Hatfield, Hertfordshire, AL9 7TA United Kingdom

*email: cRicciBonot@lincoln.ac.uk

Supplementary material


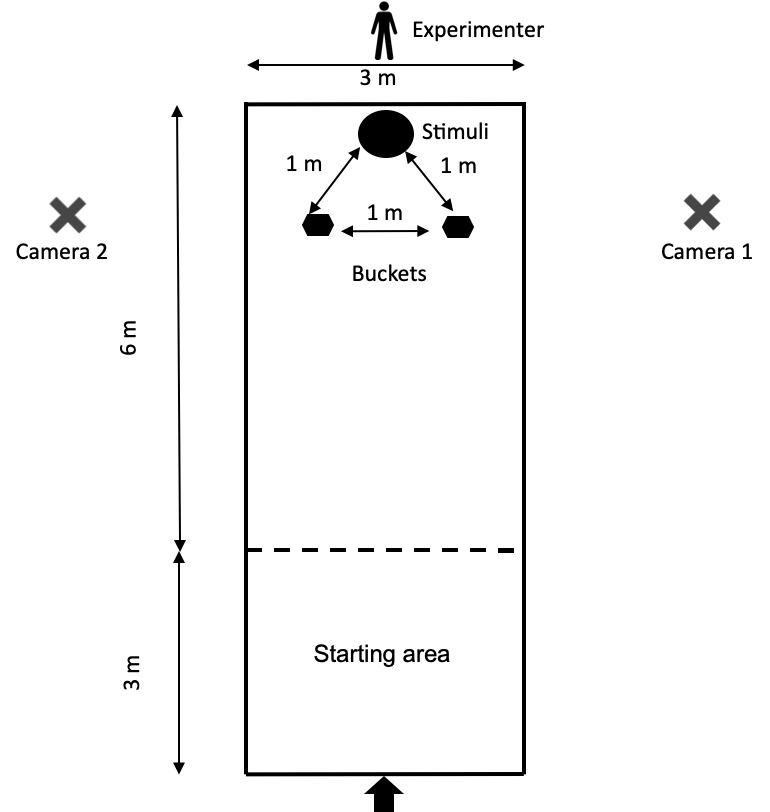


*Figure S1: Test arena for the experiments. The stimulus is a ball for the novel object test and an umbrella for the opening umbrella test*

*Table S1: Ethogram of reactivity scores (Adapted from Christensen et al., 2008 [1])*

** These reactions were allowed for habituated companion during habituation.*

| **Score** | **Reaction** | **Description** |
| --- | --- | --- |
| 0 | None* | The horse does not react to the test stimulus and chewing is not interrupted. |
| 1 | Head up* | The horse raises its head from the food container and chewing may be briefly interrupted, but the horse is not alert (see below) and does not move away from the food container. |
| 2 | Alert | The horse stands vigilant with or without elevated neck or tail, head and ears oriented towards test stimulus, chewing is interrupted and the horse may move up to 2 steps backwards or sideways away from the food container. |
| 3 | Away | The horse turns or moves 3 or more steps backwards or sideways away from the food container in response to the test stimulus, typically followed by alertness. |
| 4 | Flight | The horse turns/jumps away from the food container in a sudden movement, typically followed by trotting/galloping, alertness and possibly snorting. |
| 5 | Escape | The horse leaves the test arena. |

1. Additional analyses

1.1. Verification of observer agreement on the video scoring (reactivity score)

Inter-reliability between the two evaluators which scored the behavioural reaction of the horse to presentation of the stimulus was assessed using weighted Kappa (with the package *irr*). If the evaluators did not agree on a score, the score of the first assessor was used during data analysis.

1.2. Analysis of effect of the order of tests on the behavioural and physiological responses

We established that there was no order effect on either the behavioural and physiological responses of subjects. Cumulative link mixed models (CLMM) using the adaptive Gauss-Hermite quadrature (AGQ) (with the R-package ordinal) were used for the ordinal data relating to reactivity score, and linear mixed effect models (LMM) in the R-package lmerTest used to analyse the data on heart rate recovery. The model was the same for these two analyses, with the tests (novel object vs. umbrella) and the order of the test (1, 2, 3 or 4) fixed factors and identity of the subjects a random factor in the model for each dependent variable.

2. Additional results for study 1: Impact of familiar companion

2.1. Verification of the observer agreement on the video scoring (reactivity score)

There was high inter-observer agreement on the reaction of subjects and companions (kw = 0.961 (95% Confidence Interval (CI), 0.926 to 0.995), P < 0.0001).

2.2. Analysis of effect of the order of tests on the behavioural and physiological responses

The subject’s reactivity score and the heart rate recovery were not affected by the test order (RS, CLMM: estimate ± S.E.: −0.23 ± 0.21, P = 0.271; HRR, LMM: t-value, df: −0.62, 43.122, P = 0.534).

3. Additional results for study 2: Impact of unfamiliar companion

3.1. Verification of observer agreement on the video scoring (reactivity score)

There was high inter-observer agreement on the reaction of subjects and companions (kw = 0.975 (95% CI, 0.950 to 0.999), P < 0.0001).

3.2. Analysis of effect of the order of tests on the behavioural and physiological responses

The order of tests did not affect the subject’s reactivity score or heart rate recovery (RS, CLMM: estimate ± S.E.: −0.18 ± 0.24, P = 0.444; HRR, LMM: t-value, df: −0.15, 44.523, P = 0.884).

4. Supplementary references

1. Christensen, J. W., Malmkvist, J., Nielsen, B. L., & Keeling, L. (2008). Effects of a calm companion on fear reactions in naive test horses. *Equine Veterinary Journal, 40*(1), 46-50. (doi:10.2746/042516408X245171)
